# Schlafverhalten von Personen nach Implantation einer inversen Schulterprothese im Vergleich zu einer gesunden Kontrollgruppe: Eine explorative Querschnittsstudie

**DOI:** 10.1007/s00132-024-04487-6

**Published:** 2024-03-22

**Authors:** Melanie Manser, Vilijam Zdravkovic, Eliane Traber, Daniel Erlacher, Bernhard Jost

**Affiliations:** 1 Klinik für Orthopädische Chirurgie und Traumatologie des Bewegungsapparates, Kantonsspital St. Gallen, 9007 St. Gallen, Schweiz; 2 Institut für Sportwissenschaft Universität Bern, Bremgartenstrasse 145, 3012 Bern, Schweiz; 3 Schwalbenweg 3, 3652 Hilterfingen, Schweiz

**Keywords:** Apnoe, Schlaf, Monitoring, Schlaf, Schultertotalprothese, Schlafqualität, Gesamtschlafzeit, Apnea, sleep, Monitoring, sleep, Shoulder replacement arthroplasty, Sleep quality, Total sleep time

## Abstract

**Hintergrund:**

Personen mit Schulterpathologien berichten häufig über Schlafprobleme. Die Verbesserung der Schlafqualität ist ein Behandlungsziel der Schulterendoprothetik. Bisher ist unklar, ob veränderte Anatomie und Biomechanik bei inversen Schultertotalprothesen die Schlafqualität längerfristig beeinflussen. Zu einer zuverlässigen Einschätzung führt nebst subjektiver Bewertung die Erhebung von objektiven Schlafparametern. Mithilfe der Aktigraphie werden Körperbewegungen registriert und in aktive und inaktive Phasen eingeteilt. Dank der validen Übereinstimmung mit Wach- und Schlafphasen gelingt die Berechnung der objektiven Schlafparameter.

**Ziel der Arbeit:**

Ziele der Studie waren zu untersuchen, ob sich objektive Schlafparameter bei Personen mit inverser Schultertotalprothese („reverse total shoulder arthroplasty“ [RTSA]) ab einem Jahr postoperativ im Vergleich zu einer gesunden Kontrollgruppe unterscheiden und was die Gründe dafür sind.

**Material und Methoden:**

Die vorliegende Arbeit ist eine explorative Querschnittsstudie mit einem Messzeitpunkt. 29 Studienteilnehmende (15 RTSA-Gruppe, 14 Kontrollgruppe) erhoben während 7 Nächten mithilfe der Aktigraphie objektive Schlafparameter und Daten zur Körperlage. Der Mann-Whitney-U-Test wurde für den Mittelwertvergleich der Schlafparameter verwendet. Gründe für die Wachphasen wurden explorativ untersucht.

**Ergebnisse und Diskussion:**

Die Gruppen zeigten bei allen objektiven Schlafparametern keine signifikanten Unterschiede mit einer nahezu identischen Schlafeffizienz (*p* = 0,978). Die RTSA-Gruppe lag zu 11 % auf der operierten Seite und zu 65 % auf dem Rücken. Dies ist im Vergleich zur Kontrollgruppe mit 45 % Rückenlage knapp über dem Signifikanzniveau (*p* = 0,056). Das vermehrte Einnehmen der Rückenlage könnte atembezogene Schlafstörungen fördern und bedarf weiterer Forschung.

**Graphic abstract:**

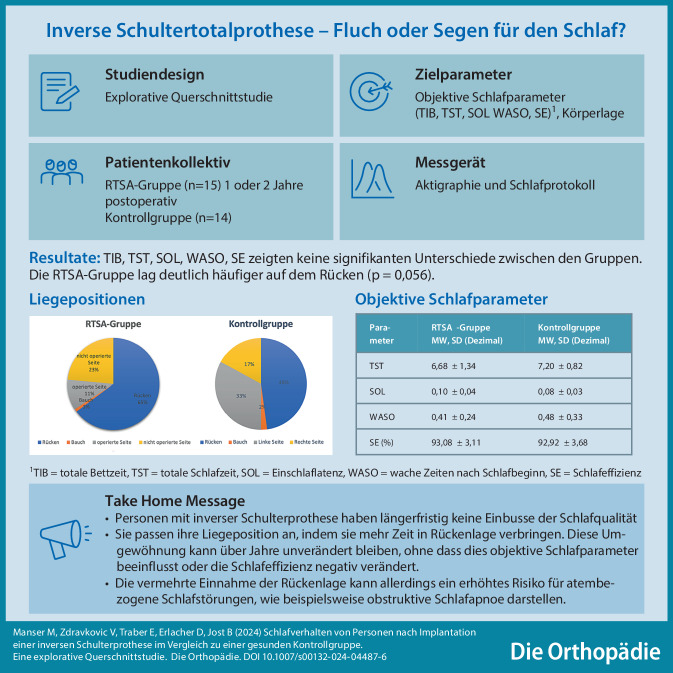

## Abstract (en)

**Background:**

Individuals with shoulder pathologies frequently report sleep problems. Improving sleep quality is a treatment focus of shoulder arthroplasty. So far, it is unclear whether altered anatomy and biomechanics in reversed total shoulder arthroplasty affect sleep quality in the long term. In addition to a subjective evaluation, a reliable assessment can be obtained by recording objective sleep parameters. With the help of actigraphy, body movements are registered and divided into active and inactive phases by means of threshold values. Thanks to the valid correspondence with waking and sleeping phases, the calculation of objective sleep parameters is successful.

**Objectives:**

The aims of the study were to investigate whether objective sleep parameters differ in persons with reversed total shoulder arthroplasty (RTSA) 1 year postoperatively compared to a healthy control group and to explore what the reasons are.

**Material and methods:**

The present work is an exploratory cross-sectional study with one measurement time point. 29 study participants (15 in the RTSA-group, 14 in the control group) collected objective sleep parameters and body position data during seven nights using actigraphy. The Mann–Whitney–U test was used for the mean comparison of sleep parameters. In addition, reasons for wakefulness were explored.

**Results and conclusions:**

The groups showed no significant differences in all objective sleep parameters with nearly identical sleep efficiency (*p* = 0.978). In the RTSA-group, 11% lay on the operated side and 65% on the back. This is just above the significance level compared to the control group with 45% in the supine position (*p* = 0.056). The increased use of the supine position could promote sleep-related medical conditions such as sleep apnoea and requires further research.

## Hinführung zum Thema

Schlafprobleme bei Personen mit Schulterpathologien sind ein weit verbreitetes Phänomen [15, 22]. Die Verbesserung der Schlafqualität ist bei gegebener Indikation eines der Behandlungsziele der Schulterendoprothetik [2]. Bisher ist unklar, ob veränderte Anatomie und Biomechanik bei inversen Schultertotalprothesen die Schlafqualität längerfristig beeinflussen, denn für eine zuverlässige Einschätzung fehlen bisher objektive Schlafparameter. In diesem Beitrag werden objektiv gemessene Schlafparameter bei Personen mit inverser Schultertotalprothese („reverse total shoulder arthroplasty“ [RTSA]) mit einer gesunden Kontrollgruppe verglichen und die Auswirkungen auf den Schlaf aufgezeigt.

## Hintergrund und Fragestellung

Personen mit Schulterpathologien zeigen aufgrund von Schmerzen und Unbehagen auf der betroffenen Seite zu liegen, eine sehr hohe Prävalenz von Einschlaf- und Durchschlafproblemen [15, 22]. Die Schlafqualität hat jedoch einen großen Einfluss auf verschiedene Gesundheitsaspekte. Sie wirkt sich auf das Hormon- und Herz-Kreislauf-System sowie das mentale Wohlergehen aus und erfüllt eine wichtige Funktion für die Gedächtniskonsolidation [3, 19]. Insgesamt sind Schlafprobleme oftmals der Hauptgrund, warum Personen mit Schulterbeschwerden eine chirurgische Beratung aufsuchen [2].

Seit der Entwicklung der modernen Schulterendoprothetik hat sich dieses Verfahren mittlerweile als eine effektive und erfolgreiche Behandlungsmethode bei Schultergelenksarthrose, irreparablen Rotatorenmanschettenrupturen oder auch Humerusfrakturen etabliert [6]. Hinsichtlich der Schulterendoprothetik existiert klare Evidenz für ein gutes Outcome, was mit reduzierten Schmerzen, verbesserter Schulterfunktion und hoher Patientenzufriedenheit einhergeht [14, 15, 22]. Weitere Studienresultate zeigen zudem auf, dass Personen nach Implantation einer Schultertotalprothese innerhalb eines Jahres über eine Verbesserung des Schlafs berichten. Die Autoren untersuchten jeweils die subjektive Schlafqualität mittels Fragebogen. [14, 22].

Ansok et al. untersuchten erstmals den Schlaf bei Personen mit Schulterpathologie mittels Aktigraphie-Geräten und erkannten, dass die subjektive Wahrnehmung von den objektiv gemessenen Schlafparametern stark abweichen kann [2]. Zudem wurde bisher bei der Untersuchung der Schlafqualität bei Personen mit Schultertotalprothese nicht zwischen einer anatomischen und inversen Schultertotalprothese (RTSA) unterschieden. Die anatomischen und biomechanischen Veränderungen bei der RTSA führen allerdings zu einer Umformung des Schulterreliefs, was wiederum Schmerzen oder ein Unbehagen hervorrufen kann, wenn Betroffene auf der Schulter liegen [10, 23]. Außerdem ist im Langzeitverlauf bei RTSA signifikant häufiger ein Außenrotationsdefizit messbar [17], was sich negativ auf die Schlafposition auswirken kann. Dies beispielsweise bei verminderter Fähigkeit den Arm in eine hohe Abduktions- und Außenrotatiosposition zu führen.

Den Goldstandard zur Erfassung der objektiven Schlafparameter stellt die Polysomnographie dar. Signaldaten verschiedener Körperfunktionen erlauben Rückschlüsse auf die Schlafphasen (Wach‑, Einschlaf‑, Tiefschlaf- und REM-Phase) [4, 7, 12]. Die Aktigraphie erkennt keine Schlafphasen, stellt aber zur Erfassung der objektiven Schlafparameter eine evidenzbasierte, kostengünstige und praktikable Alternative dar [12].

Ziele für diese Arbeit waren erstens zu untersuchen, ob sich die mit Aktigraphie objektiv gemessenen Schlafparameter bei Personen mit RTSA ab einem Jahr postoperativ im Vergleich zur gesunden Kontrollgruppe unterscheiden und zweitens zu explorieren, was mögliche Gründe für einen veränderten Schlaf bei Personen mit RTSA sind.

## Methodik

Diese explorative Querschnittsstudie mit einem Messzeitpunkt erhielt die Genehmigung durch die Ethikkommission Ostschweiz (BASEC Nr. 2021-01024).

Der primäre Outcome-Parameter war die Schlafeffizienz (SE), da dieser die Vergleichbarkeit trotz hoher intra- und interindividueller Variabilität der Schlafdauer erlaubt.

Zur Berechnung der minimalen Stichprobengröße wurde eine Power-Analyse durchgeführt. Hierzu wurde ein Unterschied der SE zwischen den Gruppen von 10 % angenommen. Die erwartete Standardabweichung von ±8,5 wurde aus der Studie von Ansok et al. [2] entnommen, bei welcher die Autoren ebenso objektive Schlafparameter mit Aktigraphie bei Personen mit Schulterpathologien erhoben. Mit einem Alpha von 0,05 und einer Power von 80 % lag die minimale Stichprobengröße bei 11 pro Gruppe (total 22).

### Rekrutierungsprozess und Stichprobe

Die Untersuchungsstichprobe bestand aus 29 Studienteilnehmenden; RTSA-Gruppe *n* = 15 (nach Defektarthropathie *n* = 10, nach Fraktur *n* = 5), Kontrollgruppe (KG) *n* = 14.

Die RTSA-Gruppe wurde aus den klinisch/radiologischen 1‑ oder 2‑Jahres-Verlaufskontrollen nach RTSA am Kantonsspital St. Gallen (KSSG) rekrutiert.

Die Einschlusskriterien waren nebst dem unterschriebenen allgemeinen Generalkonsent der Institution das Alter von mindestens 18 Jahren und das Verständnis der deutschen Sprache in Wort und Schrift. Ausgeschlossen wurden Personen mit einer diagnostizierten Schlafstörung, alle anderen Schulterprothesentypen, Schmerzen an anderen Körperstellen, Einnahme psychoaktiver Substanzen, kognitive Beeinträchtigung oder Urteilsunfähigkeit.

Die Rekrutierung der KG geschah im selben Zeitrahmen entweder aus den regulären Nachkontrollen aus anderen Sprechstunden des KSSG, oder freiwilligen Personen. Die KG wurde für Geschlecht und Alter vorsondiert, damit sie approximativ der RTSA-Gruppe entsprach.

Die Einschlusskriterien waren die gleichen wie bei der RTSA-Gruppe. Hinzu kam komplette Schmerzfreiheit, keine Schulterbeschwerden sowie keine Beweglichkeitseinschränkungen von Körperteilen. Ausgeschlossen wurden die Personen nach den gleichen Kriterien wie bei der RTSA-Gruppe.

**Figure 1:**
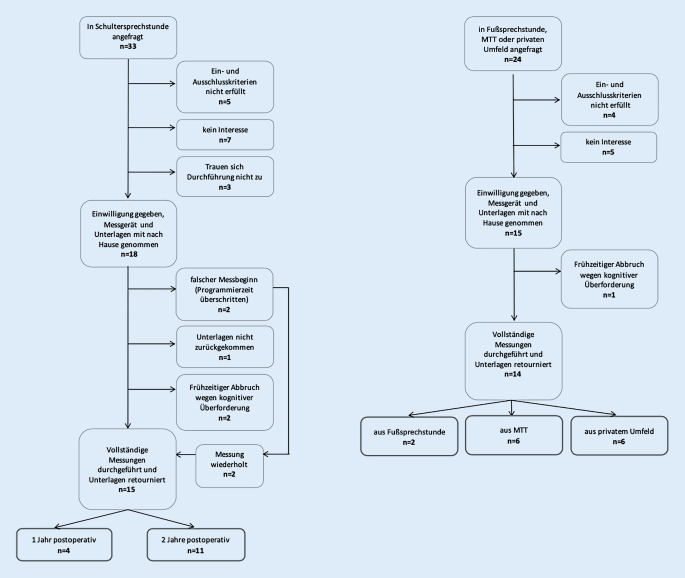

Abb. 1 zeigt den Rekrutierungsprozess.

Tab. 1 zeigt die demographischen Daten beider Gruppen.

**Table 1:** 

	RTSA-Gruppe	Kontrollgruppe (KG)	*p‑Wert*
	MW ± SD oder *n* (%)	MW ± SD oder *n* (%)	
*Alter (Jahre)*	67,93 ± 10,63	68,21 ± 8,21	0,937^a^
*BMI (kg/m*^*2*^*)*	26,37 ± 3,75	24,35 ± 3,63	0,198^a^
*Geschlecht*			
männlich	6 (40)	7 (50)	0,867^b^
weiblich	9 (60)	7 (50)	
*Nikotinkonsum*			
Nein	13 (86,7)	12 (85,8)	1^c^
Ja	2 (13,3)	2 (14,2)	
*Alkoholkonsum*			
Nein	10 (66,7)	6 (42,9)	0,36^b^
Ja	5 (33,3)	8 (57,1)	
*Beruf*			
Pensioniert	8 (53,3)	8 (57,1)	0,837^b^
Arbeitstätig	7 (46,7)	6 (42,9)	

*BMI *Body-Mass-Index,* MW* Mittelwert, *RTSA* „reverse total shoulder arthroplasty“, *SD* „standard deviation“ (Standardabweichung)

^a^Statistischer Test Mann-Whitney‑U

^b^Statistischer Test Pearson Chi^2^

^c^Statistischer Test exakter Test nach Fisher

Bezüglich der Durchführung eines Mittagsschlafs sowie der Einnahme von Schlafmedikamenten gab es keine signifikanten Unterschiede zwischen den Gruppen. Auch der Schlaf-Fragebogen B (SF-B/R) der das qualitative Schlafverhalten und Schlaferleben in den zurückliegenden 2 Wochen bewertete, zeigte keine signifikanten Unterschiede zwischen beiden Gruppen.

### Aktigraphie

Der Aktigraph, an Handgelenk oder Thorax getragen, ist ein valides Messinstrument für die Erfassung von objektiven Schlafparametern und wird häufig zur Erhebung von Schlafdaten eingesetzt, da er im heimischen Setting eingesetzt werden kann [12]. Er registriert die Körperlage und ebenso Körperbewegungen mittels Beschleunigungssensoren in drei Dimensionen (x-y-z-Achse), wobei der resultierende Wert die Beschleunigungsstärke darstellt (Einheit mg, 1 Hz, Körperlage alle 30 s).

Mithilfe eines definierten Schwellenwertes wird die Erkennung von Aktivitäts- und Ruhezyklen ermöglicht. Dank der validen Übereinstimmung dieser Zyklen mit Wach- und Schlafphasen gelingt die Errechnung der objektiven Schlafparameter [4, 16]. Daraus kann die Schlafeffizienz (SE) ermittelt werden. Dieser Wert beschreibt, wie viel der im Bett verbrachten Zeit tatsächlich geschlafen wurde. Die SE als dimensionsloses Verhältnis ermöglicht den Vergleich zwischen zwei Studiengruppen trotz hoher intra- und interindividueller Variabilität der totalen Schlafzeiten [7].

Gemäß Validierungsstudien ist die Übereinstimmung zwischen der PSG und der Aktigraphie sehr hoch und erreicht Werte von 77–91 %. Die Sensitivität (Erkennen von Schlafphasen) liegt bei 91–96 %, die Spezifität (Erkennen von Wachphasen) bei 38–54 % [4, 11, 12, 16].

**Figure 2:**
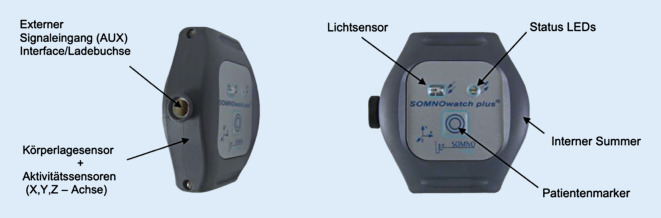

Als Messgerät für diese Studien wurden 5 Aktigraphen SOMNOwatch® (SOMNOmedics, Randersacker) verwendet (Abb. 2). Einstellungen und Aktivitätsschwelle (28 mg) wurden aus der Validierungsstudie der SOMNOwatch® mit der PSG übernommen [4]. Tab. 2 zeigt die generierten objektiven Schlafparameter.

**Table 2:** 

Abkürzung	Bedeutung	Beschreibung
TIB	„time in bed“	Gesamtdauer in Minuten vom Zeitpunkt des „Licht aus“ bis zum Zeitpunkt des „Licht an“
WASO	„wake after sleep onset“	Gesamtdauer in Minuten an Wachphasen, vom Zeitpunkt des Einschlafens bis zum Zeitpunkt des „Licht an“
SOL	„sleep onset latency“	Gesamtdauer in Minuten, die für das Einschlafen benötigt wurden, folglich die Gesamtdauer vom Zeitpunkt des „Licht aus“ bis zum Einschlafen
TST	„total sleep time“	Gesamtdauer der tatsächlich geschlafenen Zeit, folglich TIB abzüglich SOL und WASO
SE	„sleep efficiency“	Schlafeffizienz; Verhältnis von TST zu TIB

Der primäre Outcome-Parameter war die SE. Die sekundären Outcome-Parameter waren TIB, TST, SOL, WASO, Körperlage sowie Gründe für die Wachphasen.

### Schlafprotokoll

Das Schlafprotokoll (Erfassung abends und morgens) diente dazu, fehlende Messwerte aus der Aktigraphie zu kompensieren, die Angaben zur SOL zu präzisieren sowie die Wachphasen zu explorieren.

### Untersuchungsablauf

Zu Beginn wurden die ST über Studienziele, Untersuchungsablauf und verwendete Messgeräte aufgeklärt, eine schriftliche Einverständniserklärung eingeholt sowie soziodemographische Daten erhoben.

**Figure 3:**
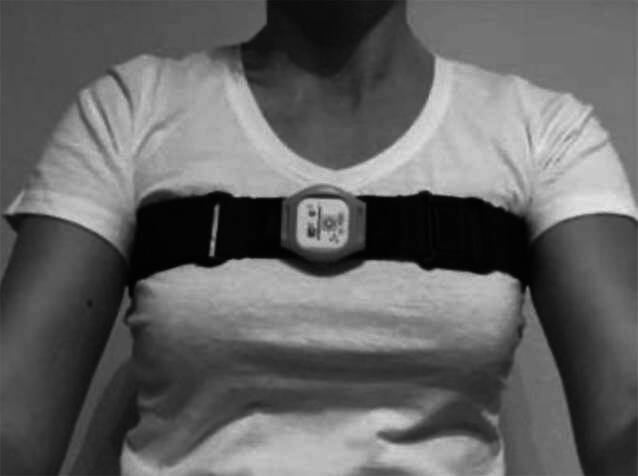

Die Messungen wurden während 7 Nächten (Ausgleich der Nacht-zu-Nacht-Variabilität [18]) mit Start am selben Abend zu Hause durchgeführt. Die Bettzeiten waren nicht vorgegeben. Die Aktigraphen wurden mittels Thoraxgurt mit Platzierung auf dem Brustbein getragen (Abb. 3). Zudem füllten die ST ein Schlafprotokoll aus. Nach Abschluss dieser Messphase schickten die ST die Studienunterlagen zurück an die Studienleiterin.

Danach wurden die Daten von der Studienleiterin auf die Domino Light Software transferiert, die Schlaf-Wach-Analyse durchgeführt und die objektiven Schlafparameter berechnet. Für die Analyse der objektiven Schlafparameter wurden alle ST inkludiert, welche im Minimum drei Nächte mit vollständigen Daten auf der Aktigraphie aufwiesen. Für die Analyse der Köperlage und für die Gründe der Wachphasen wurden alle vorhandenen Daten verwendet.

### Statistik

Die statistischen Analysen wurden mit R (R Core Team [2022]. R: A language and environment for statistical computing. R Foundation for Statistical Computing, Wien. URL: https://www.R-project.org/) durchgeführt.

Für die Analyse der kontinuierlichen Daten wurde der Mann-Whitney-U-Test gewählt. Kategoriale Daten wurden mithilfe des Pearson Chi^2^-Tests analysiert und verglichen (Darstellung in Häufigkeit *n* und %). Der exakte Test nach Fisher wurde bei Bedarf (zu erwartende Häufigkeiten < 5 in mindestens einer Zelle) als Alternative für den Pearson Chi^2^-Test ausgewählt. Für alle Analysen galt ein *p*-Wert von < 0,05 als statistisch signifikant.

## Ergebnisse

Total wurden 183 Nächte protokolliert (KG 85, RTSA-Gruppe 98). Vollständige Aktigraphie-Messnächte waren 150 vorhanden (KG 76, RTSA-Gruppe 74). Tab. 3 zeigt die objektiven Schlafparameter aus der Schlaf-Wach-Analyse. Die Resultate sind in Dezimalwerten sowie in Stunden und Minuten angegeben.

**Table 3:** 

	RTSA-Gruppe	Kontrollgruppe	*p‑Wert*
	MW, SD	MW, SD	
TIB	7,18 ± 1,45	7,78 ± 0,95	0,30
	7 h 11 min ± 1 h 27 min	7 h 47 min ± 57 min	
TST	6,68 ± 1,34	7,20 ± 0,82	0,34
	6 h 41 min ± 1 h 20 min	7 h 12 min ± 49 min	
SOL Aktigraphie	0,10 ± 0,04	0,08 ± 0,034	0,14
	6 min ± 2 min	2 min ± 2 min	
SOL Protokoll	20 min ± 16 min	19 min ±14 min	0,98
WASO Aktigraphie	0,41 ± 0,24	0,48 ± 0,33	0,70
	25 min ± 14 min	29 min ± 20 min	
WASO Protokoll	27 min ±17 min	41 min ± 60 min	0,81
SE (%)	93,08 ± 3,11	92,92 ± 3,68	0,97
Rückenlage	64,7 % ± 19 %	45,4 % ± 22 %	0,056
Bauchlage	1 % ± 1,5 %	2 % ± 3 %	0,78
Operierte Seite	10,7 % ± 12 %	–	–
Nicht operierte Seite	23,5 % ± 22,5 %	–	–

*MW* Mittelwert, *RTSA* „reverse total shoulder arthroplasty“, *SD* „standard deviation“ (Standardabweichung), *SE* „sleep efficiency“, *SOL* „sleep onset latency“, *TST* „total sleep time“, *WASO* „wake after sleep onset“

Die Resultate zeigen keine signifikanten Unterschiede zwischen den Gruppen, wobei der Vergleich der verbrachten Zeit in Rückenlage sehr knapp über dem Signifikanzniveau (*p* = 0,056) liegt.

Fünf von 12 Personen lagen während aller Messnächte nie auf ihrer operierten Seite. In der KG hingegen gab es nur eine Person, die entweder nur links oder nur rechts lag. Alle anderen Teilnehmenden aus der KG wechselten zwischen Rückenlage, linker und rechter Seite ab.

**Figure 4:**
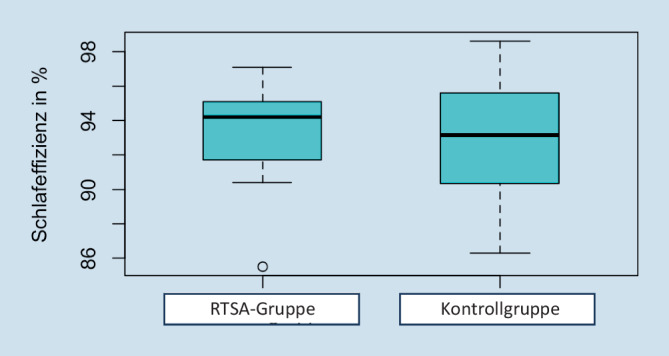

Der abgebildete Boxplot in Abb. 4 illustriert, dass der Median der SE in der RTSA-Gruppe leicht höher liegt. Die KG zeigt im Vergleich einen größeren Interquartilsabstand; die Daten sind folglich in der KG weniger konsistent.

**Figure 5:**
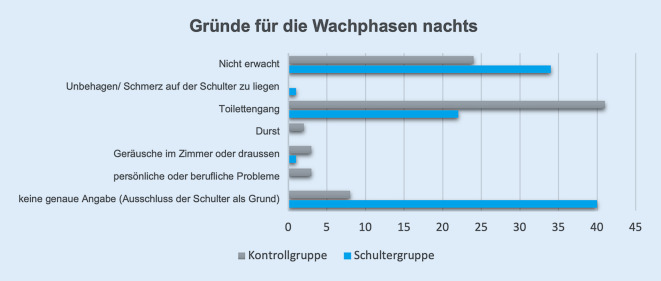

In Abb. 5 sind die Gründe für die Wachphasen dargestellt. Die x‑Achse zeigt die Anzahl der Nächte, in denen der Grund genannt wurde.

Schmerzen an der operierten Schulter wurde in einer Nacht von einer Person genannt. In der RTSA-Gruppe wurde häufig keine genauen Angaben für die Wachphasen gemacht (40 Nennungen), außer dass die Schulter jeweils nicht der Grund war. Toilettengänge machten einen großen Anteil der Wachphasen in beiden Gruppen aus.

## Diskussion

Die Resultate dieser Studie zeigen auf, dass Personen mit RTSA mindestens 1 Jahr postoperativ hinsichtlich der erhobenen objektiven Schlafparameter keine Unterschiede mehr aufweisen im Vergleich zur KG. Das bedeutet, dass Personen mit RTSA im Langzeitverlauf trotz veränderter Anatomie der Schulter objektiv keinen schlechteren Schlaf mehr zeigen im Vergleich zu Personen gleichen Alters ohne Schultertotalprothese. Für die Praxis ist dies eine wertvolle Erkenntnis, da ein markanter Teil der Personen mit Schulterproblemen im Akutstadium über große Schlafprobleme klagt [15, 22], was zum Zeitpunkt ab 1 Jahr postoperativ objektiv nicht mehr nachgewiesen werden konnte. Diese Resultate unterstützen die bisherigen Erkenntnisse zur subjektiven Einschätzung der Schlafqualität [15, 22].

Vorgängige Studien zeigten auf, dass Personen mit Schulterbeschwerden mit durchschnittlichem Schmerzwert von 7/10 (0 = keine Schmerzen, 10 sehr starke Schmerzen) auf der Numerischen Rating-Skala (NRS) die TST und die SE deutlich negativ beeinflussen [2]. In dieser Studie waren kaum mehr Schmerzen vorhanden mit einem durchschnittlichen Schmerzwert von 0,77 ± 1,11/10 auf der NRS. Dies unterstreicht den bereits mehrfach belegten großen Einfluss von Schmerzen auf die Schlafqualität [2, 13, 15, 21]. Ebenso konnten Tajika et al. in ihrer Arbeit aufzeigen, dass eine eingeschränkte Funktion der oberen Extremität mit vermehrten Schlafstörungen korreliert [20]. In der vorliegenden Studie wurde kaum über eine eingeschränkte Funktion der Schulter berichtet mit einem durchschnittlichen Wert von 0,92 ± 1,16 (0 = gar nicht eingeschränkt, 10 = sehr stark eingeschränkt).

Gründe für die Wachphasen in der Nacht waren nicht mehr die Schmerzen in der Schulter oder das Unbehagen auf der Schulter zu liegen. Andere Gründe, wie beispielsweise Toilettengänge, waren in beiden Gruppen dafür verantwortlich. Es ist bekannt, dass bei älteren Personen die Fragmentierung des Schlafs zunimmt. Einerseits durch häufigeren Tagschlaf [7] und andererseits durch die vermehrten Wachphasen nachts durch die Zunahme an Morbiditäten [8]. Ganze Nächte ohne zu erwachen wurden von der RTSA-Gruppe deutlich häufiger genannt. Es wäre möglich, dass das angewöhnte vermehrte Liegen auf dem Rücken im Langzeitverlauf zu einem ruhigeren Schlaf führt.

Die Resultate zur Körperlage dieser Studie legen dar, dass Personen mit RTSA im Schlaf mit 64,7 % deutlich länger auf dem Rücken lagen als die KG mit 45,4 %. Gemäß vorangegangener Studie, welche die Liegepositionen in der gewöhnlichen Population untersuchte, liegen die Personen durchschnittlich zu 33 % auf dem Rücken und zu 60 % auf der Seite (37 % rechts, 23 % links) [23]. Verglichen mit der vorliegenden Studie fällt auf, dass die KG mit insgesamt rund 47 % Seitenlage nah an diesen Ergebnissen war im Vergleich zur RTSA-Gruppe mit rund 34 % Seitenlage. Möglicherweise gewöhnen sich Personen mit Schulterbeschwerden automatisch ab auf der Seite zu liegen. Durch das Einnehmen der Rückenlage reduziert sich der Druck in der Schulter und somit die Schmerzen. Die Resultate könnten darauf hinweisen, dass die Personen diese Umgewöhnung auch 1 oder 2 Jahre postoperativ nicht wieder verändert haben. Diese Hypothese unterstützt auch die Tatsache, dass 5 von 12 Personen während allen Messnächten nie auf ihrer operierten Seite geschlafen haben und dies dennoch nicht als störend oder einschränkend empfanden.

Das vermehrte Schlafen in Rückenlage kann aus Sicht der Schlafforschung dennoch problematisch werden. Denn das wichtigste Merkmal schwerer obstruktiver respiratorischer Ereignisse ist, dass sie in Rückenlage auftreten. Eine Person mit obstruktiver Schlafapnoe beispielsweise weist in Rückenlage eine ungünstige Lage der Atemwege mit eingeschränkter Funktion der Atemwegsmuskulatur auf [9]. Die vermehrte Einnahme der Rückenlage bei Personen nach RTSA sollte folglich im Fokus weiterer Untersuchungen bleiben.

## Limitationen

Die Studie hat Limitationen. Folgende Punkte müssen bei der Interpretation der Ergebnisse berücksichtigt werden:

- Stichprobengröße: Die Stichprobe war relativ klein. Deswegen wurde eine Post-hoc-Power-Analyse durchgeführt, um Beta-Fehler zu überprüfen. Diese zeigte, dass bei einer Stichprobe von *n* = 14 pro Gruppe und dem SE-Mittelwert der Kontrollgruppe von 92,9 %, die SE-Mittelwerte der RTSA-Gruppe < 89 % (Grenzwert) mit ausreichend Power untersuchbar waren. Somit liegt die Unsicherheitsgrenzwert innerhalb von 3,9 %, was klinisch nicht relevant ist. Aufgrund der Stichprobengröße war keine Subanalyse nach Pathologie (Defektarthropathie versus Fraktur) möglich.
- „Selection bias“: Möglicherweise haben eher diejenigen Personen mitgemacht, die zufrieden mit ihrer inversen Schulterprothese waren. Andererseits könnten in der Kontrollgruppe vor allem diejenigen mitgemacht haben, die schlecht schlafen und diese Studie ihr Interesse und ihre Motivation geweckt hat, dies objektiv zu untersuchen.
- Reliabilität der Messnächte: Es waren zwar 7 Nächte vorgesehen, bei der Betrachtung der absolvierten Messnächte inklusive der „missing data“ gab es aber Unterschiede zwischen den Studienteilnehmenden, was die Inkludierung der Wochenendnächte anbelangt. Aili et al. [1] zeigten in ihrer Arbeit auf, dass die Reliabilität größer war, wenn nur die Nächte während der Woche inkludiert wurden.
- Positionierung des Aktigraphen am Thorax: In der Schlafmedizin sind keine klaren Grenzwerte für die SE definiert [7], dennoch ist bekannt, dass die SE durch die Zunahme der Fragmentierung des Schlafs bei älteren Personen abnimmt und somit Werte über 90 % in dieser Altersklasse als hoch einzustufen sind [5]. Dies könnte darauf zurückgeführt werden, dass der Aktigraph am Thorax getragen wurde. Dies ist zwar die empfohlene Position, wenn auch die Körperlage miterfasst wird. Das Tragen am Thorax könnte dennoch zu geringeren Aktivitätswerten (zu wenig gutes Erkennen der Wachphasen) und somit höheren SE-Werten geführt haben. Außerdem kann die Positionierung ventral am Thorax dazu beigetragen haben, dass die Bauchlage weniger häufig eingenommen wurde. Wenn die Ergebnisse dieser Studie mit anderen Studienresultaten verglichen werden, muss der Trageort des Aktigraphen berücksichtigt werden.

## Fazit für die Praxis

- Die Untersuchung der Schlafqualität und die Erhebung von objektiven Schlafparametern ist ein Feld mit sehr hoher intra- und interindividueller Variabilität.
- Die Resultate dieser Studie verbessern und konkretisieren dennoch die Einschätzung zur Schlafqualität nach RTSA („reverse total shoulder arthroplasty“), was ein relevanter und positiver Aspekt bei der Patientenaufklärung darstellt.
- Personen mit Schulterbeschwerden können ihre Gewohnheiten zu den Liegepositionen verändern und passen sich auch längerfristig an, indem sie mehr Zeit in Rückenlage verbringen.
- Diese Umgewöhnung kann über Jahre unverändert sein, ohne dass dies die Schlafqualität oder die SE („sleep efficiency“) negativ beeinflusst.
- Aus Sicht der Schlafforschung muss die vermehrte Einnahme der Rückenlage und das damit verbundene Risiko für atembezogene Schlafstörungen, wie beispielsweise obstruktive Schlafapnoe, im Fokus der Forschung bleiben.
